# Benefits of Iron Chelators in the Treatment of Parkinson’s Disease

**DOI:** 10.1007/s11064-021-03262-9

**Published:** 2021-03-01

**Authors:** Xiaoyan Zeng, Hedi An, Fei Yu, Kai Wang, Lanlan Zheng, Wei Zhou, Yiwen Bao, Jie Yang, Nan Shen, Dongya Huang

**Affiliations:** 1 Department of Neurology, East Hospital, Tongji University School of Medicine, Shanghai, 200120 China; 2 Department of Infectious Diseases, Shanghai Children’s Medical Center, Shanghai Jiao Tong University School of Medicine, Shanghai, 200127 China

**Keywords:** Parkinson’s disease, Iron deposition, Ferroptosis, Iron chelator, Neuroprotective effect

## Abstract

As a novel discovered regulated cell death pattern, ferroptosis has been associated with the development of Parkinson’s disease (PD) and has attracted widespread attention. Nevertheless, the relationship between ferroptosis and PD pathogenesis is still unclear. This study aims to investigate the effect of iron overload on dopaminergic (DA) neurons and its correlation with ferroptosis. Here we use nerve growth factor (NGF) induced PC12 cells which are derived from pheochromocytoma of the rat adrenal to establish a classical PD in vitro model. We found significantly decreased cell viability in NGF-PC12 cell under ammonium ferric citrate (FAC) administration. Moreover, excessive intracellular iron ions induced the increase of (reactive oxygen species) ROS release as well as the decrease of mitochondrial membrane potential in PC12-NGF cells. In addition, we also found that overloaded iron can activate cell apoptosis and ferroptosis pathways, which led to cell death. Furthermore, MPP-induced PD cells were characterized by mitochondrial shrinkage, decreased expression of glutathione peroxidase 4 (Gpx4) and ferritin heavy chain (FTH1), and increased divalent metal transporter (DMT1) and transferrin receptor 1 (TfR1) expression level. In contrast, Lip-1 and DFO increased the expression level of GPX4 and FTH1 compared to MPP-induced PD cell. In conclusion, we indicated that overloaded intracellular iron contributes to neurons death via apoptosis and ferroptosis pathways, while DFO, an iron chelator, can inhibit ferroptosis in order to protect the neurons in vitro.

**Supplementary Information:**

The online version contains supplementary material available at 10.1007/s11064-021-03262-9.

## Introduction

Parkinson’s disease (PD) is one of the most common neurodegenerative diseases. Its main clinical manifestations include motor symptoms (bradykinesia, rigidity, resting tremor, postural instability) and non-motor symptoms (impairment of olfaction, depression, sleep disorder). The most prominent pathological features of PD are the loss of dopaminergic (DA) neurons in the substantia nigra (SN) and the abnormal accumulation of α-synuclein (α-Syn) [1, 2]. Researches found that the major motor symptoms of PD occur due to a deficit of dopamine and the degeneration of the nigro-striatal pathway that release dopamine caused an imbalance of excitatory (acetylcholine) and inhibitory (dopamine) neurotransmitters in the region [3]. Yet, due to its complex pathology, there is still no unified theory to explain the degeneration and death of DA neurons in PD.

Recent studies have suggested that high levels of iron play a critical role in the occurrence and development of neurodegenerative diseases, by activating oxidative stress and other processes [4, 5]. In addition, it has been found that iron metabolism dysfunction has a key role in development and progression of neurodegenerative diseases. In PD patients, iron–melanin interactions promote dopaminergic neuron degeneration [4–6]. Moreover, in our previous study, we found a significantly higher iron content in the SN of PD patients compared to healthy controls; in addition, the amount of iron was related to the clinical symptoms of patients by using quantitative susceptibility mapping (QSM) [7] Additionally, studies have shown that iron has an important protective role in the immune system of vertebrates. For example, Gallin et al. found that lactoferrin secreted from neutrophils is capable of sequestering iron ions in the body fluid circulation and inhibit the growth of the fungal pathogen *Aspergillus fumigatus* [8]. Moreover, Goetz et al. discovered that lipocalin 24p3 binds to the *E. coli* iron carrier and inhibits iron absorption by *E. coli* [9].

So far, many studies have confirmed that iron overload in the brain is closely associated with α-synuclein accumulation, oxidative stress, mitochondrial dysfunction, neuroinflammation, and ubiquitin protease system disorder, which all mediate the death of PD DA neurons [10–20]. However, it is still not clear whether high iron is one of the main causes of damage mechanisms or secondary changes caused by other injury factors. In 2012, a novel regulated cell death pathway-ferroptosis was discovered by studying the pancreatic cancer; the process is characterized by loss of intracellular glutathione (GSH) and the inactivation of glutathione peroxidase 4 (Gpx4) [21]. Lipid oxides in cells produce a large amount of lipid reactive oxygen species (ROS) through a Fenton’s reaction process in the presence of a large number of ferrous ions, which in turn damage the functions of mitochondria and ubiquitin proteasomes, leading to cell death [22–24]. Currently, the mode of neuronal death remains unclear. It is believed that apoptosis may be one of the causes of extensive DA neuron degeneration in PD. Yet, the characteristics of generally enhanced neuroinflammation and chronic degeneration found in autopsy of PD patients cannot be explained only by apoptosis. In addition, drugs targeting apoptosis, such as caspase inhibitors, MKL inhibitors, etc., have not achieved the expected results in clinical practice.

Ferroptosis, a novel cell death mode triggered by massive lipid peroxidation is associated with degeneration of spinal motoneurons and midbrain neurons. The pathogenesis of many neurological diseases has been associated with ferroptosis, including Huntington’s disease (HT), Alzheimer’s disease (AD), PD, stroke, and brain trauma, etc. [21–25]. Researched found that activation of ferroptosis results in the non-apoptotic destruction of certain cancer cells, while inhibition of this process may protect neurons from neurodegeneration [21]. In the process of PD dopaminergic neuron degeneration, some phenomena similar to the characteristic pathological changes of ferroptosis, such as loss of GSH by SN iron deposition, increased lipid peroxidation and ROS, and mitochondrial dysfunction were observed. [22, 24]. Treatment with *N*-acetylcysteine (NAC), a precursor of GSH, can rescue neuronal damage of PD in vivo [23, 26, 27]. In addition, the use of iron chelators has been shown to significantly improve the motor symptoms of PD in patient (phase II clinical trials) and animal models [22, 23]. These results suggest that ferroptosis pathway may have a key role in the development of PD.

Although a large number of studies have suggested that iron overload does participate in the pathogenesis of PD and has an important role in the whole process, it is still not clear whether iron overload is the main cause of PD or a pathological phenomenon during PD progression. Furthermore, it’s molecular mechanisms associated with neural cells damage remain unexplored. The following study examined the effects of iron overload on DA neurons and it’s correlation with ferroptosis.

## Materials and Methods

### Cell Culture

SH-SY5Y cells were cultured in DMEM/F12 (Hyclone SH30023.01) medium supplemented with 10% fetal bovine serum (FBS, life 26140079) and 1% penicillin–streptomycin (Pen/Stre, life 15140122). PC12 cells were grown in RPMI-1640 (Hyclone SH30809) medium supplemented with 10% heat inactivated horse serum (HS, Gibco 16050122), 5% fetal bovine serum (FBS, life 26140079), and 1% penicillin–streptomycin (Pen/Stre, life 15140122). All cells were incubated in a humidified atmosphere containing 5%CO_2_/95% air at 37 °C. The medium was replaced every day, until the cells reached 80–90% confluency.

PC12 cells, derived from rat adrenal medulla pheochromocytoma were cultured in 0.01% poly-lysine (PLL; Sigma, 25988-60-3) coated6-well plate (Corning) with (3–5) × 10^4^ cells/well. The 6-wellplate was placed in a cell culture incubator at 37 °C, 5% CO_2_ and saturated humidity overnight, at which time the differentiation day 0 was recorded, and the cell morphology was photographed under an inverted microscope. The cells were differentiated by the addition of nerve growth factor (50 ng/ml NGF; Sigma, N0513) to the serum free medium in a final concentration of 100 ng/ml. The state of differentiation was followed by simple phase-contrast microscopy every other day. After 6–8 days, an estimated 80–90% of the cells were differentiated into cells with a neuronal-like phenotype. The differentiation of the cells was repeated five times, all resulting in the same degree of differentiation.

Six days after treatment with NGF, the cells were additionally treated with iron overload, chelating compounds, or ferroptosis inhibitor Liproxstatin-1. Iron overload was induced by FAC (Sigma, F5879, concentrations ranged from 0 to 1000 μM). After incubation for 72 h, culture solutions containing different concentrations of DFO and Liporaxstatin-1 (Lip-1) were added.

### Cell Viability

Cell death rate was determined by the MTT (Sigma M2128) assay. CCK-8 kit (Beyotime C0042) was used to measure the cell proliferation index. Additionally, Annexin V-FIRC/PI Apoptosis Detection Kit (Yeasen 40302ES60) was used to detect cell death, following the manufacturer’s protocol.

### ROS Assessment

DCFH-DA working solution was diluted in serum-free medium according to the ROS Kit instructions (Nanjing Jiancheng E004). The intracellular ROS release level was measured by flow cytometry, microplate reader, and fluorescence microscope, respectively. For the mirocplate reader detection, the Ex of 485 nm and Em of 525 nm were used.

### JC-1 Detection

The JC-1 staining working solution was prepared according to the specifications of the manufacture for the use of the Mitochondrial Membrane Potential (JC-1) Detection Kit (Beyotime C2006). The potential was measured by flow cytometry and fluorescence microscope, respectively. The excitation wavelength was 488 nm, while the emission wavelength was FL1 (525 ± 20 nm) and FL2 (585 ± 20 nm).

### Western Blot

Total proteins were obtained using protein isolation kits (BeyotimesP0013J) according to the manufacture’s instruction. The protein extracts were subjected to SDS-PAGE and then transferred to PVDF membranes. After blocking by BSA for about 1 h, proteins were incubated overnight at 4 °C with primary antibodies: TH (Abcam ab6211); DMT1 (Abcam ab55735); TfR1 (Abcam ab1086); FPN (Abcam ab78066); FTH1 (EPR18878) (Abcam ab183781); Caspase-3 (Abcam ab90437); GPX4 (Abcam ab125066); ACSL4 (Abcam ab155282). Next day, the blots were washed and incubated 1 h with horseradish peroxidase (HRP)-conjugated secondary antibodies: Goat Anti-Rabbit IgG H&L (Abcam ab6721) and Goat Anti-Mouse lgG H&L (Abcam ab6789). After washing the blots three times with TBST, the blots were visualized with an enhanced chemiluminescence detection system (*Amersham Pharmacia Biotech*). Samples were analyzed in triplicates and the measurements were averaged and used as one individual data point for statistical analysis. Quantification was done by densitometric analysis using Beta actin (Abcam ab210083) as an internal control.

### Q-PCR

Total cellular RNA from cell lysates was extracted by using TRIzol™ Reagent (Invitrogen™ 15596026) according to manufacturer’s instruction. Following extraction, RNA was treated with RNase-free DNase I (Roche, IN, USA) according to manufacturer’s protocol. RNA quality and quantity were analyzed spectrophotometrically by using NanoDrop-2000c spectrophotometer (Thermo Scientific, MA, USA) and electrophoretically by using 0.8% v/w agarose gel in 0.5× Tris–acetate (TAE) buffer. Each sample was performed in triplicates, while non-reversed RNA and water served as negative controls. Data were normalized to Beta actin. All qPCR reactions were performed using Prime Script™ RT reagent Kit with gDNA Eraser (TakaRa RR047A). Relative expression levels of the genes mentioned in Supplementary Table 1 were calculated from 2^−ΔCt^ values to the house- keeping gene [hypoxanthine phosphoribosyltransferase 1 (Hprt1) or b-actin] and normalized by control to obtain 2^−ΔΔCt^ (Table 1).

**Table 1:** qPCR primers

	Forward primer	Reverse primer
DMT1	5′-ATAGCAGCAGCCCCCATG-3′	5′-AGGCCCGAAGTAACATCCAA-3′
TFR1	5′-AGTGGTCGCTGGGTGGATT-3′	5′-CCTTCAGGCATACAGCTCAATTG3′
FPN	5′-GGTGGTGGCAGGCTCTGT-3′	5′-TTTGAACCACCAGGGACGTC-3′
Caspas-3	5′-CCACACTGATTTCTCCCGATGT-3′	5′-CGCACATCCACCCAGTTCTT-3′
ACSL4	5′-TATGGGCTGACAGAATCATG-3′	5′-CAACTCTTCCAGTAGTGTAG-3′
FTH1	5′-TGATGTGGCTTTGAAGAAC-3′	5′-GCGTCTCAATGAAGTCAC-3′
GPX4	5′-CCCAGGGCCACTTACTTTCC-3′	5′-AGGACTTTTCCTTGGGTCGG-3′
TH	5′-TCGGAAGCTGATTGCAGAGA-3′	5′-TTCCGCTGTGTATTCCACATG-3′
Beta actin	5′-ATCGTGGGCCGCCGCCCTAGGCA-3′	5′-TGGCCTTAGGGTTCAGAGGGG-3′

### Transmission Electron Microscope

PC12-NGF cells were washed with PBS and treated with 0.2 ml trypsin (25300062, Thermo Fisher Scientific) for 1–2 min. Cells were then transferred to 1.5 ml centrifuge tube, washed by prechilled PBS once, centrifuged, and fixed by 2.5% glutaraldehyde (in 0.1 M PBS) for 1 h at room temperature followed by further fixation at 4 °C overnight. After post-fixation by 1% osmium tetroxide (in 0.1 M PBS) for 1 h, cells were dehydrated with graded ethanol solutions (30, 50, 70, 80, 95, and 100%) and sodium sulfate anhydrous treated acetone. Specimens were infiltrated sequentially with a 1:1 mixture of acetone and EPON resin for 1.5 h, and 100% EPON resin overnight. Specimens were then embedded in embedding molds and polymerized for 48 h in an oven at 60 °C. Thin sections (60–90 nm) were cut using a Leica ultramicrotome, mounted on copper grids, and stained with uranyl acetate and lead citrate. Transmission electron microscopy images were captured using FEI Tecnai G2 Spirit Twin. All image data shown were representative of at least three randomly selected fields from at least three independent experiments.

### Statistical Analysis

Statistical Package for Social Scientists (SPSS) version 20.0 was used for all statistical analyses. The Kruskal–Wallis test was used to look for effects of the various treatments on the parameters of interest. Statistical significance was determined by two-tailed Student’s *t*-test for two groups or Bonferroni test. Image J software was used for image analysis. Data are shown as mean ± SEM or SD as indicated in figure legends. Data was obtained from at least three independent experiments. In all cases, *P* value < 0.05 (*P < 0.05, **P < 0.01, ***P < 0.001) was considered statistically significant.

## Results

### Effects of FAC on PC12-NGF Cell Viability

As previously described, we used NGF to induce PC12 cells to get a sympathetic neurons-like cell. After treatment with NGF (50 ng/ml), PC12 cells developed obvious synapses, and the volume of cell bodies increased and changed from circular/elliptical to polygonal/diamond shape (Supplementary Fig. 1 A). In addition, we used a western blot to analyze the expression levels of dopaminergic neuron-specific marker tyrosine hydroxylase (TH), and found that TH expression levels were significantly increased (Supplementary Fig. 1 B).

**Fig. 1:**
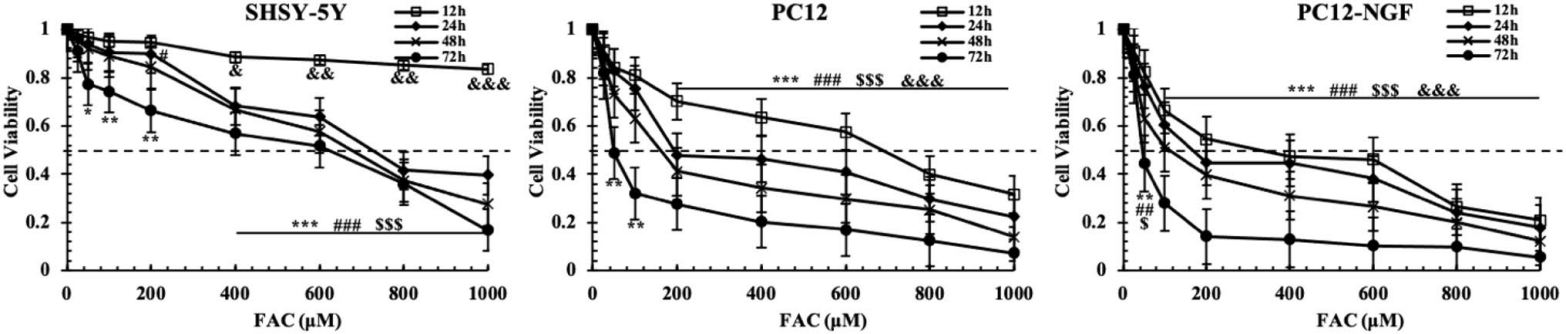
Effects of FAC on cell viability. Cell viability was measured by MTT assay; the experiment was run in triplicate for each group. The Bonferroni method & Dunnett’s T3 method were used to compare the differences between groups. The test level is α = 0.05; X μMvs 25 μM; ^&^24 h, ^$^48 h, ^#^72 h, *96 h

Through MTT assay we found that the cell viability rate of SH-SY5Y, PC12 and PC12-NGF cells showed an decreasing trend after FAC treatment in a concertation-dependent and time-dependent manner. Among the three cells, PC12-NGF viability was most affected by FAC. The PC12-NGF cells viability decreased to 44.4% after treatment with 50 μM FAC for 72 h, when the FAC concentration rise to 1000 μM the cell death rate of PC12-NGF cell was close to 5.6% (Fig. 1). Obviously, this result demonstrated that FAC has drastically cytotoxicity, especially in sympathetic neurons. However, what kind of cell damage did FAC induced and how this process happened.

### FAC Induced Iron-Overload Leads to Oxidative Stress of PC12-NGF Cell

Studies found that FAC administration can lead to intracellular iron-overload, which then caused an increase of ROS release [28–32]. By western blot and q-PCR we observed that with the increase of FAC concentration and the prolongation of treatment time, the expression level of iron metabolism proteins DMT1 and TfR1 were significantly increased at both the protein and mRNA level, which indicated that the intracellular iron ion content of PC12-NGF cells significantly increased after FAC treatment. At the same time, we observed that the expression of TH was negatively correlated with that of DMT1 and TfR1. Low concentration of FAC (50 M) significantly affected the activity of PC12-NGF cells, and the cytotoxic effect of FAC became more obvious with the prolongation of the action time (S Fig. 1 A, B).

**Fig. 2:**
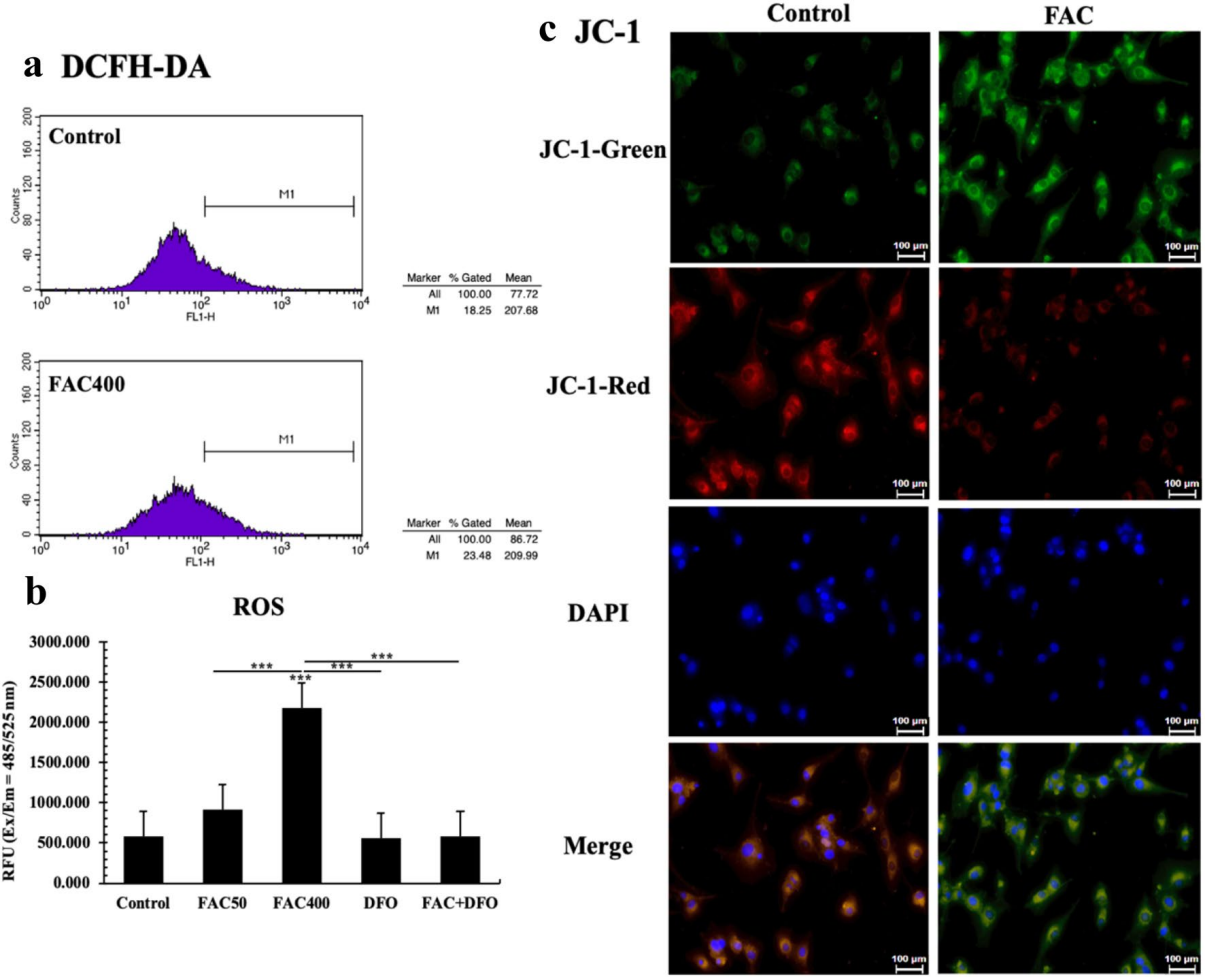
FAC induced oxidative stress. **a** Flow cytometry was used to measure ROS level; **b** Using microplate reader to detect each group’s relative fluorescence intensity; **c** Changes of mitochondrial membrane potential under FAC treatment. The relative intensity of red/green fluorescence after treatment of JC-1 in each group was observed under fluorescence microscope. The green fluorescence enhancement indicated that the mitochondrial membrane potential was decreased. The scale was 100 μm. All experiments were repeated three times and averaged. The difference between each group was compared with Bonferroni method and/or Dunnett’s T3 method. The test level is α = 0.05; *P < 0.05; **P < 0.01; ***P < 0.001

Subsequently, we measured the release of ROS by DCFH-DA, and used JC-1 to detect mitochondrial membrane potential changes. Using flow cytometry, we observed that intracellular ROS of PC12-NGF cells treated with 400 μM FAC for 72 h was significantly increased (FAC400 vs Control, *P* < 0.001) (Fig. 2a). Similar results were observed under microplate reader analysis (Ex = 485 nm, Em = 525 nm) (Fig. 2b). Next, we used the membrane-permeant JC-1 dye to monitor mitochondrial health. Red fluorescence indicated that the mitochondrial membrane potential was high, while green fluorescence indicates that the mitochondrial membrane potential was decreased. Briefly, we found higher green fluorescence in the FAC treatment compared with that of control group and FAC-DFO group (Fig. 2c), which suggested mitochondrial damage in cell treated with FAC. These above evidences indicated that FAC treatment may induce mitochondrial function damage by increasing intracellular oxidative stress levels.

### FAC Induced Iron-Overload Activates Apoptosis and Ferroptosis of PC12-NGF Cell

**Fig. 3:**
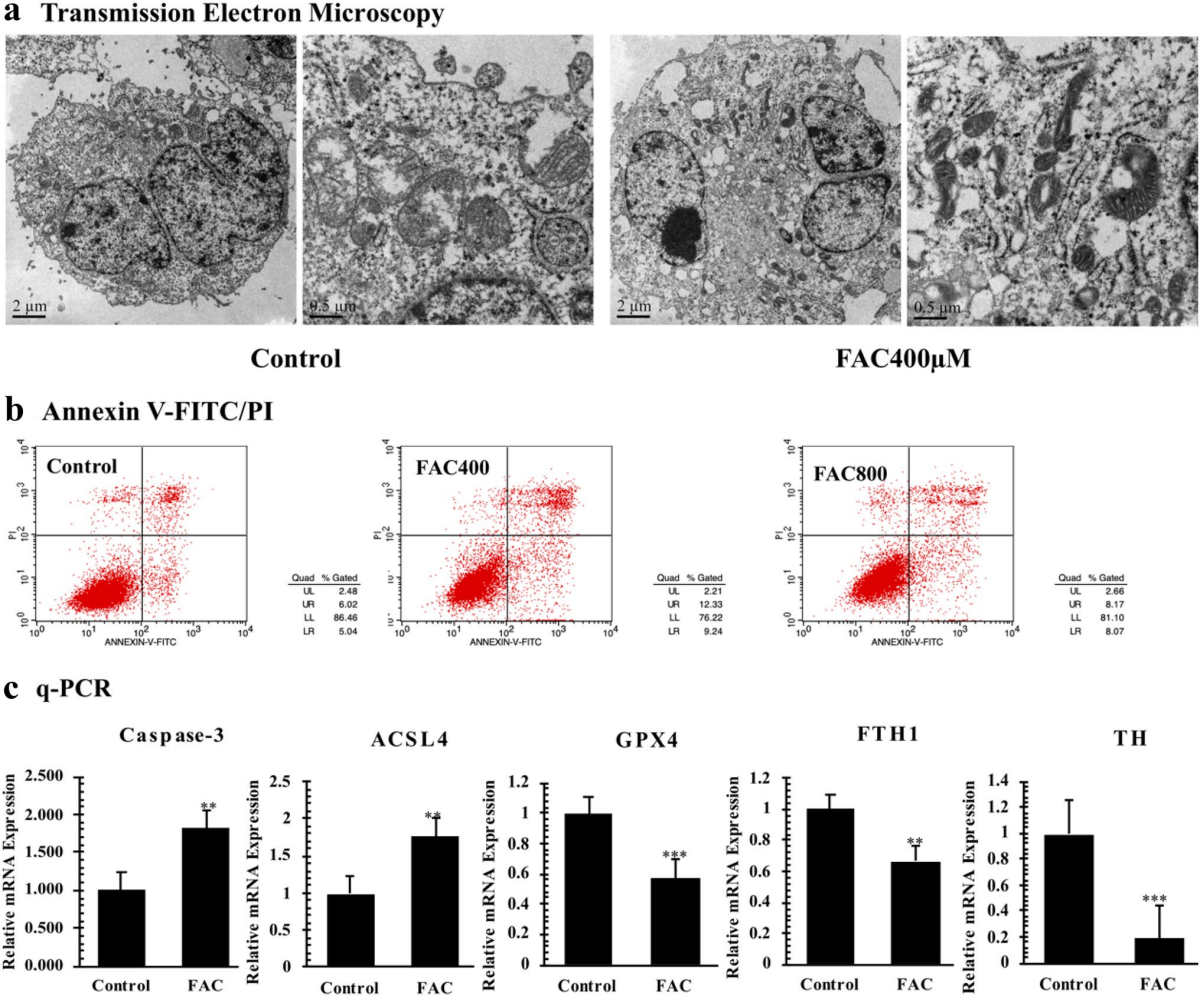
Subcellular morphology changes. **a** Transmission Electron Microscope. Mitochondrial exhibited characteristic morphology changes similar to ferroptosis, like condensed mitochondrial membrane densities, reduction of mitochondrial inner membrane folds (cristae); FAC activates Apoptosis in PC12-NGF cells. **b** Annexin V-FITC/PI kit was used to assay cell apoptosis rate. **c** The mRNA expression level of ferroptosis-related factors in PC12-NGF cells under FAC stimulation. Each test was repeated three times and averaged. The difference between each group was compared with Bonferroni method and/or Dunnett’s T3 method. The test level is α = 0.05;*P < 0.05; **P < 0.01; **P < 0.001

In order to figure out the concrete role of FAC in PC12-NGF cells mitochondria-induced cell death, the subcellular structure of PC12-NGF cell after FAC treatment was observed by transmission electron microscopy. It was observed that the volume of mitochondrial became smaller and its membrane showed a double-tracked sign similar to the characteristic change of ferroptosis. Besides, we also saw some apoptosis classical morphology changes (Fig. 3a). Then we use Annexin V-FITC/PI apoptosis kit to measure cell apoptosis. The nucleus was counter stained with DAPI dye, and the fluorescence intensity of each group was observed under an inverted fluorescence microscope. The number of apoptosis significantly increased under the action of medium/low concentrations of FAC (25–400 μM) compared to that of control group. However, when the FAC concentration was 800 μM, the number of early and late apoptotic cells were significantly reduced and the cell survival rate was decreased (Supplementary Fig. 3). After that, flow cytometry was used to detect Annexin V-FITC/PI double fluorescently labelled apoptotic cells, and results showed that high concentrations of FAC (800 μM) did inhibit the occurrence of apoptosis to a certain extent (FAC800*VS* Control, *P* < 0.01; Fig. 3b). The above results indicated that in addition to inducing apoptosis through damage to mitochondrial function, FAC certainly causes cell death through some other pathways. As mentioned before, through transmission electron microscopy, drastic morphology changes assembly to ferroptosis were observed in FAC stimulated PC12-NGF cells (Fig. 3a). Consequently, we used q-PCR to detect the expression level of ferroptosis associated factors ACSL4, FTH1, GPX4, and apoptosis key protein Caspase-3. The results showed a lower expression of GPX4 and FTH1, and a higher expression of ACSL4 in FAC-treated groups compared with that of control group. In addition, the key factors of apoptosis pathway Caspase-3 were significantly increased in the FAC-treated cells (Fig. 3c). According to our results, we believed that FAC induced iron-overload damaged PC12-NGF cells mainly through ferroptosis and apoptosis pathway, especially the ferroptosis process.

### DFO Rescues FAC Induced Iron-Overload and Ferroptosis in PC12-NGF Cells

**Fig. 4:**
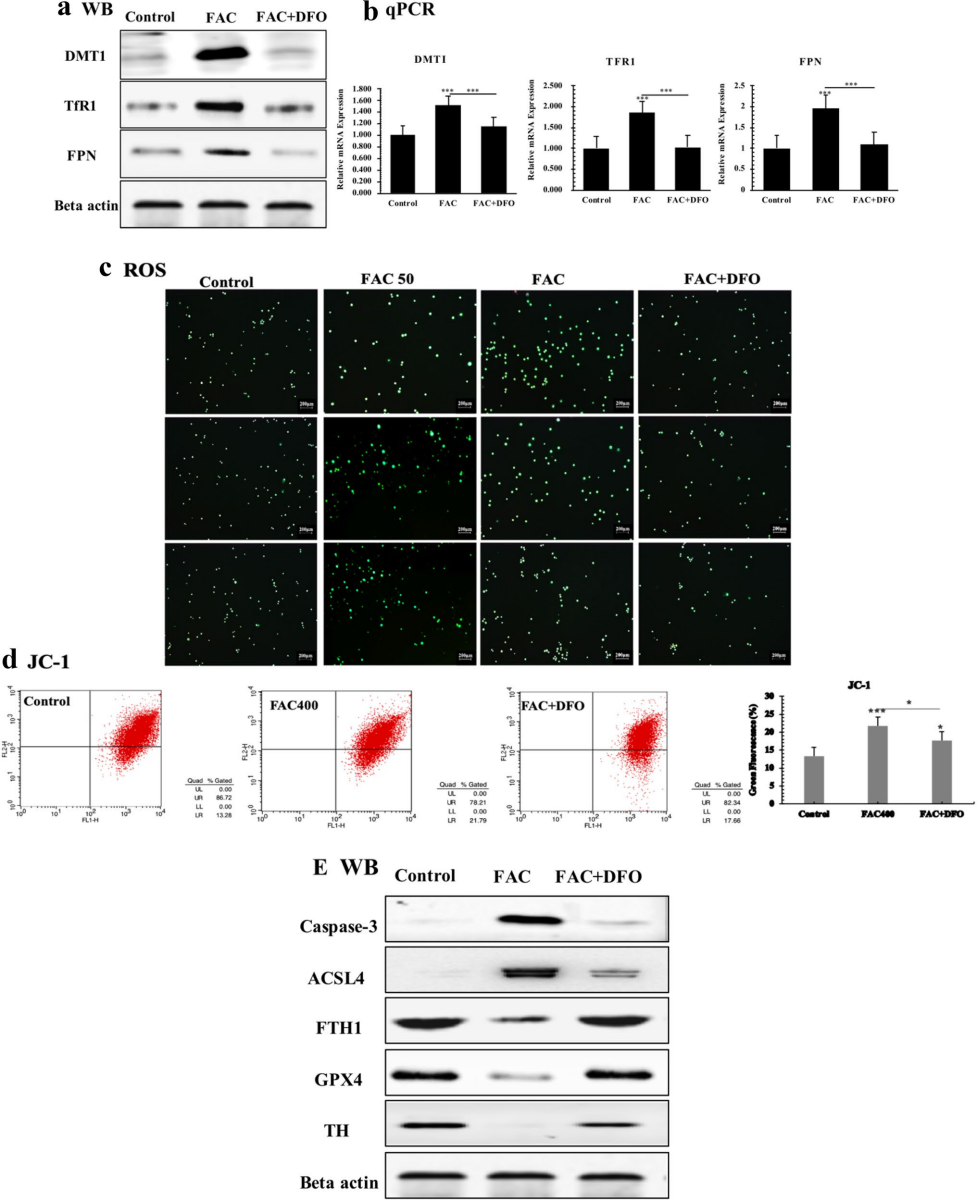
DFO rescues FAC induced iron overload. **a** Western blot; beta actin was used as an internal reference. **b** The real-time quantitative PCR analysis. DFO rescues FAC induced oxidative stress. **c** The fluorescence intensity of each group of cells stained with DCFH-DA was observed under a fluorescence microscope. The relative fluorescence intensity was calculated by Image J software, and the green fluorescence enhancement represented an increase in intracellular ROS. The scale was 200 μm. **d** Flow cytometry was used to detect the relative intensity of green fluorescence after treatment wih JC-1 of each group. DFO rescues FAC activated ferroptosis. **e** Western blot of ferroptosis-related proteins in PC12-NGF cells under FAC stimulation. All experiments were repeated three times and averaged. The Bonferroni method and Dunnett’s T3 method were used. The test level is α = 0.05; *P < 0.05; **P < 0.01; **P < 0.001, vs Control

DFO is a well-known iron chelator, here we use DFO to treat FAC administrated PC12-NGF cells. By western blot and qPCR, a significantly increased expression level of iron metabolism-related factors like DMT1, FPN and TfR1 were observed. After DFO treatment, the expression level of all these proteins returned to a relatively normal level **(**F4 A, B**)**. In order to clarify if DFO could rescue FAC induced oxidative stress in PC12-NGF cells, we measured the releasement of ROS by DCFH-DA. Intracellular ROS fluorescence intensity of PC12-NGF cells treated with 50 μM and 400 μM FAC for 72 h revealed significant enhanced green fluorescence (FAC50 vs Control, *P* < 0.05; FAC400 vs Control, *P* < 0.001) under inverted fluorescence microscopy. As the iron concentration increased, the fluorescence intensity increased. After administration of the iron chelator DFO, the ROS level decreased and returned to normal levels (Fig. 4c) Next, we used flow cytometry to measure the changes of mitochondrial membrane potential, and found that DFO treatment drastically reduced mitochondrial membrane potential compared to that of FAC induced group (Fig. 4d). These results demonstrated that iron chelator DFO can efficiently rescue FAC stimulated oxidative stress of PC12-NGF cells. As our previous results showed that FAC can activates ferroptosis pathway the same time when induced oxidative stress. We may consider that if DFO can inhibit PC12-NGF cells ferroptosis by reduce intracellular oxidative stress. By western blot we observed a lower expression of GPX4 and FTH1, and a higher expression of ACSL4 in FAC-treated groups compared with that of control group and DFO treatment group. In addition, the key factors of apoptosis pathway Caspase-3 were significantly increased in the FAC-treated cells. After administration of DFO, the expression level of Caspase-3 was significantly decreased, but was still higher than that of the control group (Fig. 4e).

### DFO and Liproxstatin-1 Decreased the ROS Release Level in PD In Vitro Model

**Fig. 5:**
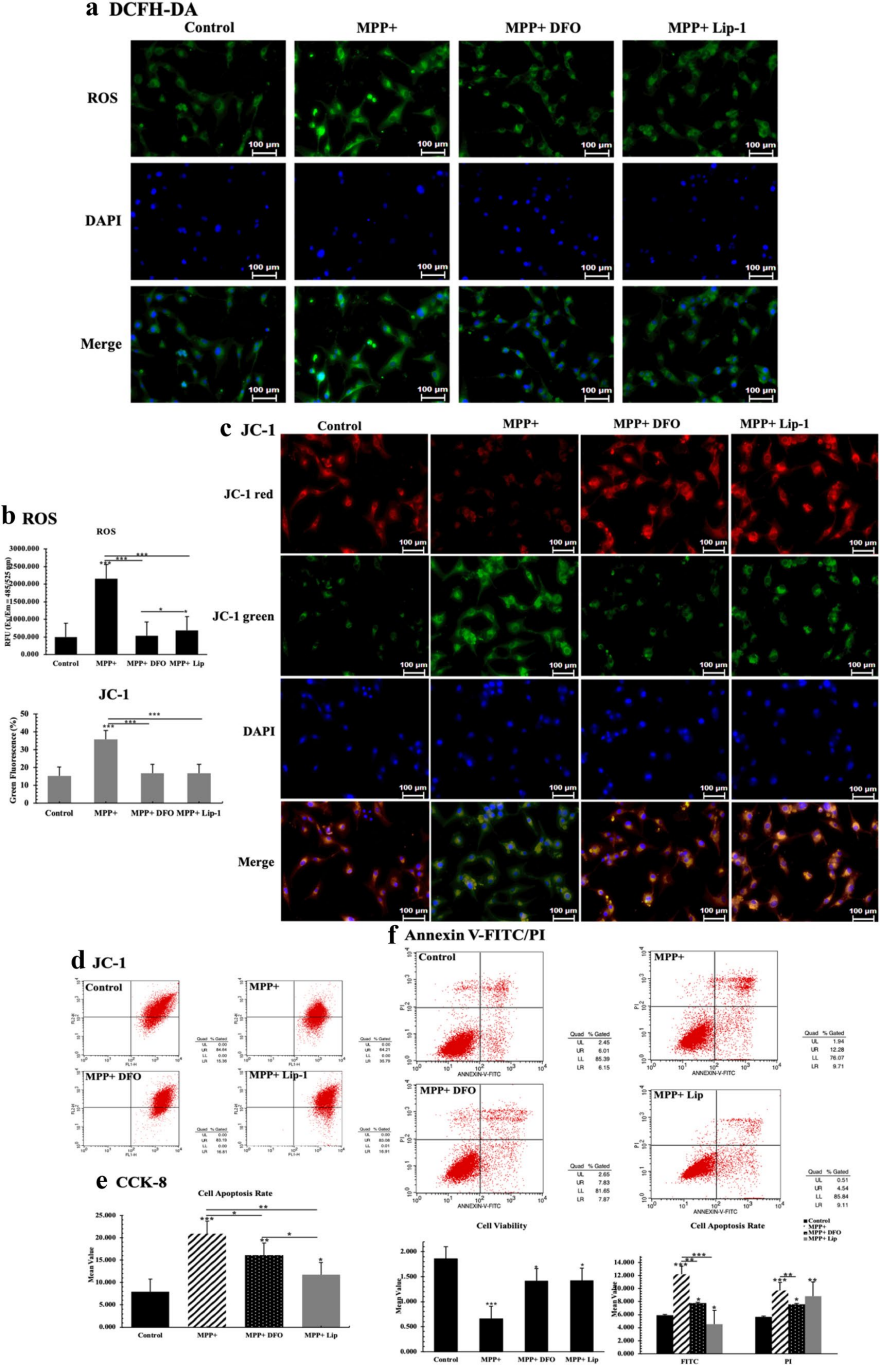
DFO reduced cellular iron overload of PD in vitro model. **a** The fluorescence intensity of each group of cells stained with DCFH-DA was observed under a fluorescence microscope. The relative fluorescence intensity was calculated using Image J software. The scale bar equals to 100 μm. **b** Using microplate reader to detect each group’s relative fluorescence intensity. **c** Detected mitochondrial membrane potential in PD cell model by JC-1 probe. The scale bar equals to 100 μm. **d** Using flow cytometry to measure mitochondrial membrane potential. Cell viability. **e** CCK-8 assay was used to analyze cell mortality; **f** cell apoptosis rate was measured by flow cytometry under Annexin V-FITC/PI kit. All experiments were repeated three times and averaged. The Bonferroni method and Dunnett’s T3 method were used. The test level is α = 0.05; *P < 0.05; **P < 0.01; **P < 0.001

Previously, our team found elevated iron deposition in PD patients SN region [7]. Moreover, many recent studies suggest that ferroptosis pathway may play a critical in the pathogenesis of PD [22–27]. We use MPP+ which is a typical PD in vitro model inducer to establish a PD cell model. After MPP+ administration, intracellular oxidative stress levels of DA neurons were significantly increased and its cell viability decreased compared with that of control group. After incubation with DCFH-DA, the fluorescence of the groups treated with iron chelator DFO and the ferroptosis pathway inhibitor Liproxstatin-1 (Lip-1) respectively were significantly decreased compared with MPP+ induced group (*P* < 0.05; Fig. 5a). In addition, the relative fluorescence intensity of ROS in each group was detected by microplate reader. Similar results were observed, and the ROS release levels of DFO-treated group decreased more than that of Lip-1-treated group (*P* < 0.05; Fig. 5a, b). At the same time, we used the JC-1 probe to detect mitochondrial membrane potential; we observed that DFO and Lip-1 both increased mitochondrial membrane potential and decreased intracellular oxidative stress (Fig. 5c, d). After that, we used CCK-8 kit to detect cell viability. The results showed that compared with the decreased intracellular oxidative stress levels, the cell viability of the treated group was significantly higher than that of the MPP+ induced model group (*P* < 0.001; Fig. 5e).

Furthermore, Annexin V/FITC apoptosis assay showed that both DFO and Lip-1 significantly reduced the apoptotic rate of PD cell model. Among them, DFO seems to have a more pronounced inhibition of early apoptosis (FITC) (Fig. 5f; Supplementary Fig. 4). Inhibition of apoptosis by DFO and Lip-1 does not appear to be sufficient to increase cell viability as much as compared to the increase in cell viability. Lip-1 is an inhibitor of ferroptosis, which primarily protects cells by inhibiting the occurrence of ferroptosis. Thus, we hypothesized that DFO is also likely to block the intracellular ferroptosis pathway.

### DFO Inhibited Ferroptosis Pathway to Protect Cells from Damage in PD In Vitro Model

**Fig. 6:**
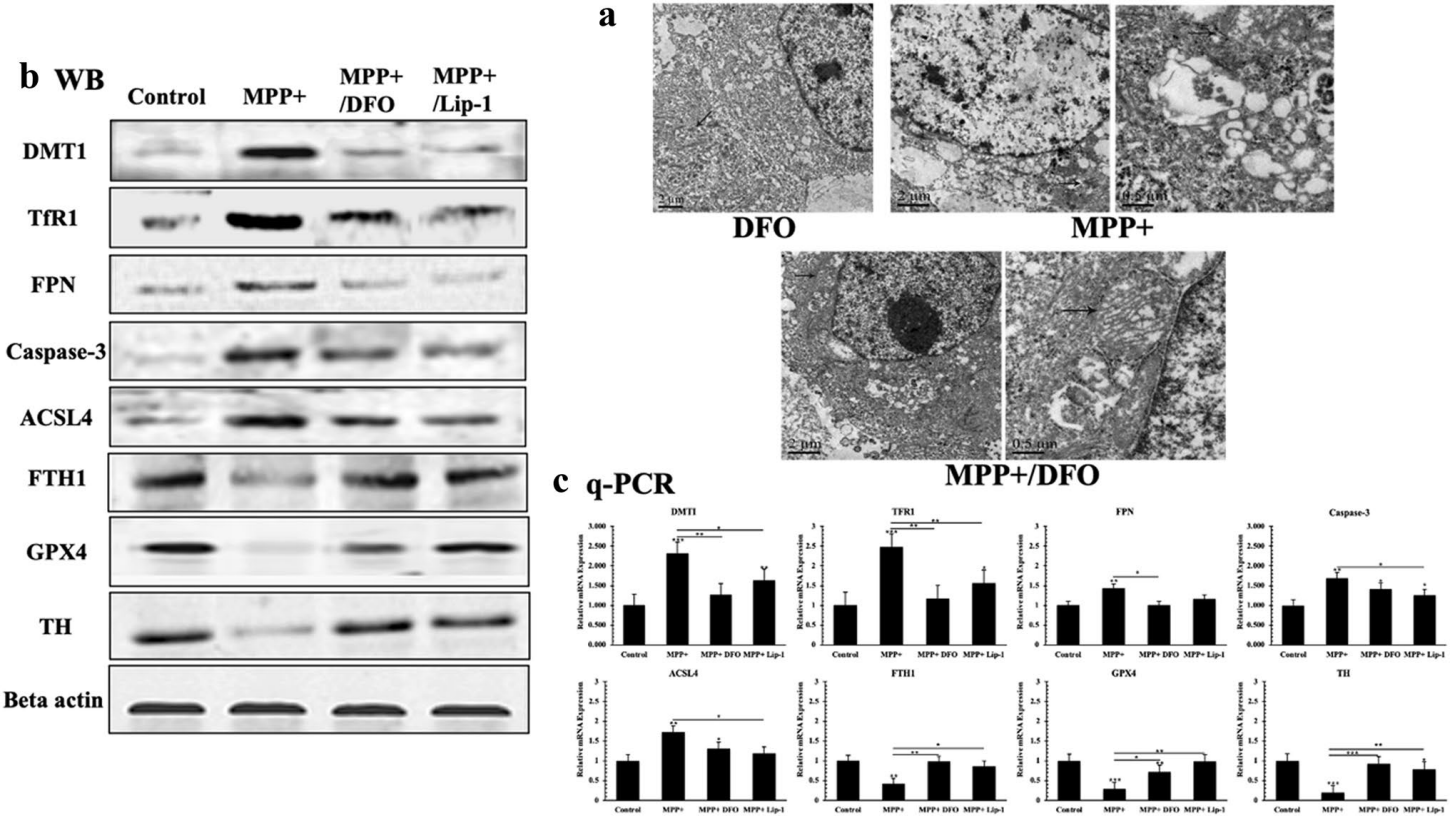
Changes of subcellular morphology. **a** Mitochondrial exhibited characteristic morphology changes similar to ferroptosis, like condensed mitochondrial membrane densities, reduction of mitochondrial inner membrane folds (cristae); the expression of TH, DMT1, TfR1, FPN, Caspase-3, ACSL4, FTH1, GPX4 in different groups. **b** Western blot analyzes; beta actin was used as an internal reference. **c** The real-time quantitative PCR was used to compare the mRNA expression levels of TH, DMT1, TfR1, FPN, Caspase-3, ACSL4, FTH1, Gpx4. All experiments were repeated three times and averaged. The Bonferroni method and Dunnett’s T3 method were used. The test level is α = 0.05; *P < 0.05; **P < 0.01; **P < 0.001, vs Control

We have previously observed an increased oxidative stress levels and cell death rate in PC12-NGF cells treated with MPP and administration compared with that of control group. Using an electron microscopy, we observed that some MPP+ induced cells showed morphological changes similar to ferroptosis (Fig. 6a). Western blot and Q-PCR analysis showed that compared with MPP+ induced model group DMT1, TfR1, and ferroptosis related factors such as GPX4 and FTH1 were significantly increased in the Lip-1 treatment group, and the expression level of ACSL4 was decreased. In addition, the expression level of the apoptotic key factor Caspase-3 was significantly increased after MPP+ administration. After DFO chelated off a large amount of intracellular free iron ions, the expression of iron metabolism related factors DMT1 and TFR1 was significantly decreased, while, the expression level of GPX4 and FTH1 was significantly increased, and the expression of ACSL4 was significantly decreased. Although we observed a decreased expression of capase-3, it was still significantly increased compared with MPP+ induced model group (Fig. 6b, c).

## Discussion

Our current data showed that FAC intervention could significantly affect cell growth and proliferation in a dose- and time dependent manner, which suggested that the cytotoxic effect of FAC is a chronic process. In this study, we found that among SH-SY5Y, PC12 and PC12-NGF cells, PC12-NGF cells are the most sensitive one to FAC. SH-SY5Y cells are derived from human neuroblastoma cell lines and have moderate TH activity, while PC12 cells have high TH activity. After inducing cell differentiation by NGF, PC12-NGF cells can acquire sympathetic neuron characteristics similar to DA neurons biochemical properties. There are many researches indicated increased iron ions in many neurodegenerative diseases [28, 29]. Similarly, our previous clinical study on PD patients showed that iron deposits are mainly concentrated in the SN primarily compared to other midbrain nuclei, like globus pallidus, caudate, and dental nucleus [7]. The above results indicated that the cytotoxicity of iron overload is a chronic accumulation process with certain cell specificity, among which midbrain DA neurons are most sensitive to the chronic cytotoxicity of iron ions.

In the present study, western blot and Q-PCR were used to detect the iron metabolism related factors DMT1, TfR1 and FPN in PC12-NGF cells. The results showed that these factors were highly expressed in FAC-treated cells, proving that iron ions can increase in PC12-NGF cells treated with FAC. Moreover, ROS release assessment revealed intracellular high oxidative stress levels and decreased mitochondrial membrane potential in those cells, suggesting that iron-dependent ROS accumulation can cause neuronal mitochondrial dysfunction, which in turn leads to neuronal death. The following apoptosis assays showed decreased apoptotic rate of PC12-NGF cells with the increased concentration of iron ions, but the overall cell survival rate continuously decreased, which suggested that there must be some other cell death pathways other than apoptosis.

Ferroptosis is a newly discovered cell death pattern that is activated by the accumulation of lipid peroxides [27], which can be blocked by iron chelators. In the absence of mechanisms to alleviate iron-related damage, the massive accumulation of intracellular ROS causes ferroptosis in cells, which differs from other cell death patterns in biochemistry and morphology [30–32]. Recently, studies found that [Ca2+] induced ROS release could also lead to a form of programmed cell death that is identical to ferroptosis [33, 34]. Although current research on the initiation mechanism of ferroptosis has achieved certain results, the exact role of iron in ferroptosis is still not clear. Glutathione peroxidase 4 is considered to be a key factor of ferroptosis [33–36]. Studies have shown that inhibition of GPX4 leads to accumulation of intracellular lipid peroxides, which ultimately causes ferroptosis [37]. The expression of acyl-CoA synthetase long chain family member 4 (ACSL4) that is considered to be one of the major driving factors for ferroptosis [38], was significantly down-regulated in cells with ferroptosis resistance (such as LNCaP and K562 cells) compared to those ferroptosis-sensitive cells (such as HepG2 and HL60 cells). Other studies suggested that ACSL4-mediated production of 5-hydroxyeicosatetraenoic acid (5-HETE) can promote ferroptosis [39–41]. In this study the expression of GPX4 and FTH1 were significantly decreased, while the expression of ACSL4 significantly increased in FAC-treated cells compared to that in control groups (Fig. 6b, c). In addition, we observed some morphological changes similar to ferroptosis under transmission electron microscope (Figs. 3a, 6a). All these results indicated that direct intervention of FAC can cause iron overload status in PC12-NGF cells, which impairs mitochondrial function and causes apoptosis. With the increased iron content, a large amount of ROS accumulates, apoptosis rate decreases, and ferroptosis is activated, eventually causing a large number of nerve cell deaths.

In this study, we found a high expression of TfR1 and DMT1, and a low expression of FTH1 in cells treated with MPP compared with that of control groups, thus suggesting that intracellular free iron ions are accumulated during MPP injury. At the same time, the low expression of ferroptosis key factor GPX4 was detected. Gpx4 protein can inhibit lipid peroxidation of cell membrane, reduce the production of intracellular lipid ROS, and inhibit the occurrence of ferroptosis [27]. Down regulated GPX4 can increase cellular ferroptosis sensitivity. Intracellular ferrous ion accumulation and imbalance of antioxidant system are the two major mechanisms of ferroptosis that mediate the production of lipid peroxides [42, 43]. MPP+ could reduce GPX4 expression and increase ACSL4 expression. The long-chain fatty acid acyl-CoA ligase 4 encoded by the ACSL4 gene is essential for lipid oxidative metabolism and is involved in the regulation of lipid metabolism processes caused by numerous arachidonic acids (AA). Studies have confirmed that ACSL4 is a key factor required for lipid peroxidation after GPX4 inactivation [37]. These results indicated that MPP+ are most probably activated through the ferroptosis pathway and thereby cause damage to DA neurons. Our experiments revealed that DFO and Lip-1 could reduce intracellular oxidative stress level, protect mitochondrial function and reduce cell apoptosis rate by chelating excessive intracellular free iron ions. In addition, we observed that DFO and Lip-1 can up-regulated expression level of Gpx4, reduce expression of ACSL4, and inhibit the occurrence of intracellular lipid peroxidation. Lip-1 is a classical ferroptosis pathway inhibitor [30] that blocks intracellular lipid peroxidation. As for that DFO exhibits neuroprotective effects similar to lip-1 in MPP+ damaging dopaminergic neurons, we hypothesized that DFO can effectively alleviate the damage of iron overload on cells by reducing intracellular free ferrous ion levels and blocking ferroptosis.

## Limitations

This study has few limitations. (1) Our data showed that iron overloads can activate apoptosis and ferroptosis in PC12-NGF cells. However, the specific pathological changes and mechanisms still remain unclear. (2) The time-dependent and concentration-dependent effects of iron-damaged PC12-NGF cells need to be further analyzed in detail. (3) The protective effect of DFO on PC12-NGF cells could be the result of multiple pathways, including apoptosis, necrosis, ferroptosis and some other cell death modes; it is necessary to further examine which one has the main role in this protective effect of DFO on PC12-NGF cells. (4) This study did not include animal experiments. In the future, we plan to construct a PD animal model to explore the inherent relation between iron overload and ferroptosis in vivo.

## Conclusion

The main findings of this study are: (1) iron accumulation induces cytotoxicity in neural-like cells in a dose- and time- dependent manner; (2) PC12-NGF cell is one of the most sensitive cells to the toxic effect of iron; (3) both apoptosis and ferroptosis pathway are activated in PC12-NGF cells under FAC administration; (4) activated ferroptosis may be one of the mechanisms of MPP+ induced PD in vitro model; (5) iron chelator DFO could inhibit ferroptosis and have a protective role in PD cell model.

## Supplementary Information

Below is the link to the electronic supplementary material.

Supplementary file1 (XLSX 61 KB)

Supplementary file2 (XLSX 226 KB)

Supplementary file3 (XLSX 58 KB)

Supplementary file4 (XLSX 24 KB)

Supplementary file5 (XLSX 430 KB)

Supplementary file6 (XLSX 140 KB)

Supplementary file7 (XLSX 10 KB)

Supplementary file8 (XLSX 49 KB)

Supplementary file9 (DOCX 14983 KB)

## Data Availability

Data transparency.

## Abbreviations

ACSL4: Acyl-CoA synthetase long chain family member 4
AD: Alzheimer’s disease
BBB: Blood brain barrier
BCECs: Capillary endothelial cells
CCK-8: Cholecystokinin-octopeptide
CFS: Cerebrospinal fluid barrier
DA: Dopaminergic neurons
DAPI: 4′6-Diamidino-2-phenylindole
DCFH-DA: 2′7-Dichlorodi-hydrofluorescein diacetate
DFO: Desferrioxamine
DMSO: Dimethyl sulphoxide
DMT1: Divalent metal transporter 1
FAC: Ferric ammonium citrate
FBS: Fetal calf serum
FITC: Fluorescein isothiocyanate
FPN: Ferroportin
FTH1: Ferritin heavy chain
Gpx4: Glutathione peroxidase 4
GSH: Glutathione
HS: Horse serum
IRP: Iron related proteins
Lip-1: Liproxsatin-1
LRRK2: Leucine-rich repeat kinase 2
MPP+: 1-Methyl-4-phenylpyridinium
MTT: Thiazolyl blue tetrazolium bromide
NAC: *N*-acetylcysteine
NADPH: Nicotinamide adenine dinucleotide phosphate
NGF: Nerve growth factor
NTBA: Non-transferrin bound iron
PBS: Phosphate buffer solution
PC12: Pheochromocytoma derived cell line
PD: Parkinson’s disease
PI: Propidium iodide
PINK 1: PTEN-induced putative kinase 1
Q-PCR: Quantitative real time polymerase chain reaction
QSM: Quantitative susceptibility mapping
ROS: Reactive oxygen species
TfR1: Transferrin receptor 1
TH: Tyrosine hydroxylase
UPS: Ubiquitin protease system
WB: Western blot
