# Supplementary material for: Benefits of Iron Chelators in the Treatment of Parkinson’s Disease
Source: Neurochem Res. 2021 Mar 1;46(5):1239–51. doi: 10.1007/s11064-021-03262-9 (PMC8053182; doi:10.1007/s11064-021-03262-9)
Supplement: Supplementary file 9 — Supplementary file9 (DOCX 14983 KB) [file 11064_2021_3262_MOESM9_ESM.docx]

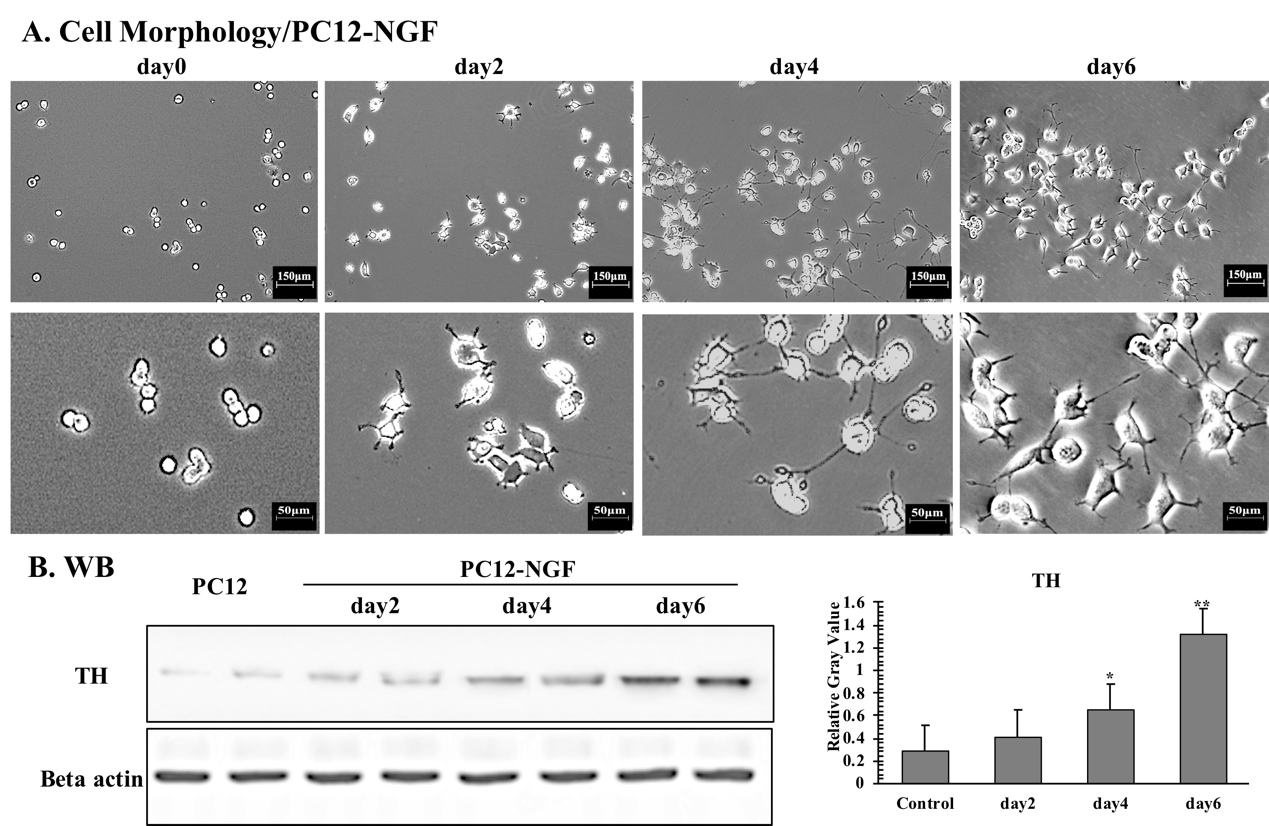
**Supplementary Figure 1**

**S Fig 1. NGF Differentiated PC12 cells*.* A:** *Cell morphology changes were observed under a light microscope;* **B:** *Western blot semi-quantitative analysis of the expression level of DA neuron-specific maker tyrosine hydroxylase (TH); Beta-actin was used as an internal reference. Bonferroni and Dunnett’s T3 tests were used to compare the grey value differences between each group. Each set of grey value was measured three times and averaged. The test level is α=0.05,* ********P<0.05;* ******** *P<0.01;* ******** *P<0.001.*


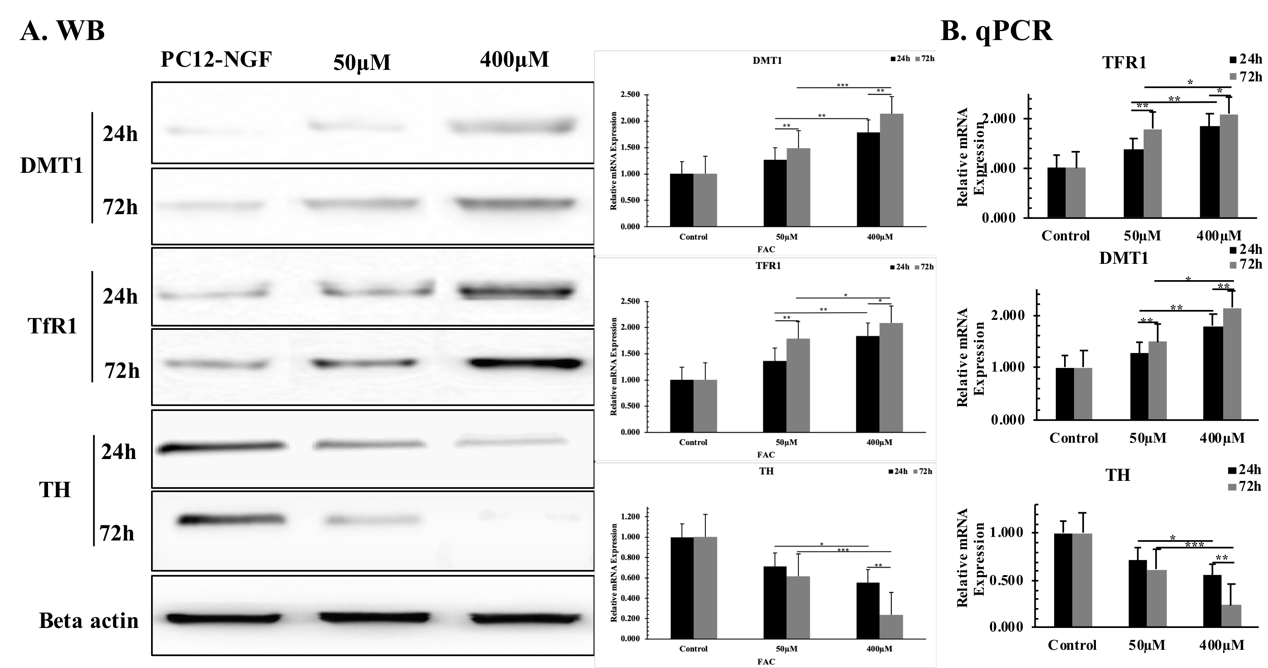
**Supplementary Figure 2**

***S Fig 2. FAC induced iron overload in PC12-NGF cells. A:*** *Western blot semi-quantitative and* ***B:*** *q-PCR analysis of the expression level of DMT1, TfR1and dopaminergic neuron-specific maker tyrosine hydroxylase (TH); Beta-actin was used as an internal reference. Bonferroni and Dunnett’s T3 tests were used to compare the gray value differences between each group. Each set of gray value was measured three times and averaged. The difference between each group was compared with Bonferroni method and/or Dunnett’s T3 method. The test level is α=0.05，*P<0.05; **P<0.01; **: P<0.001*

**Supplementary Figure 3**


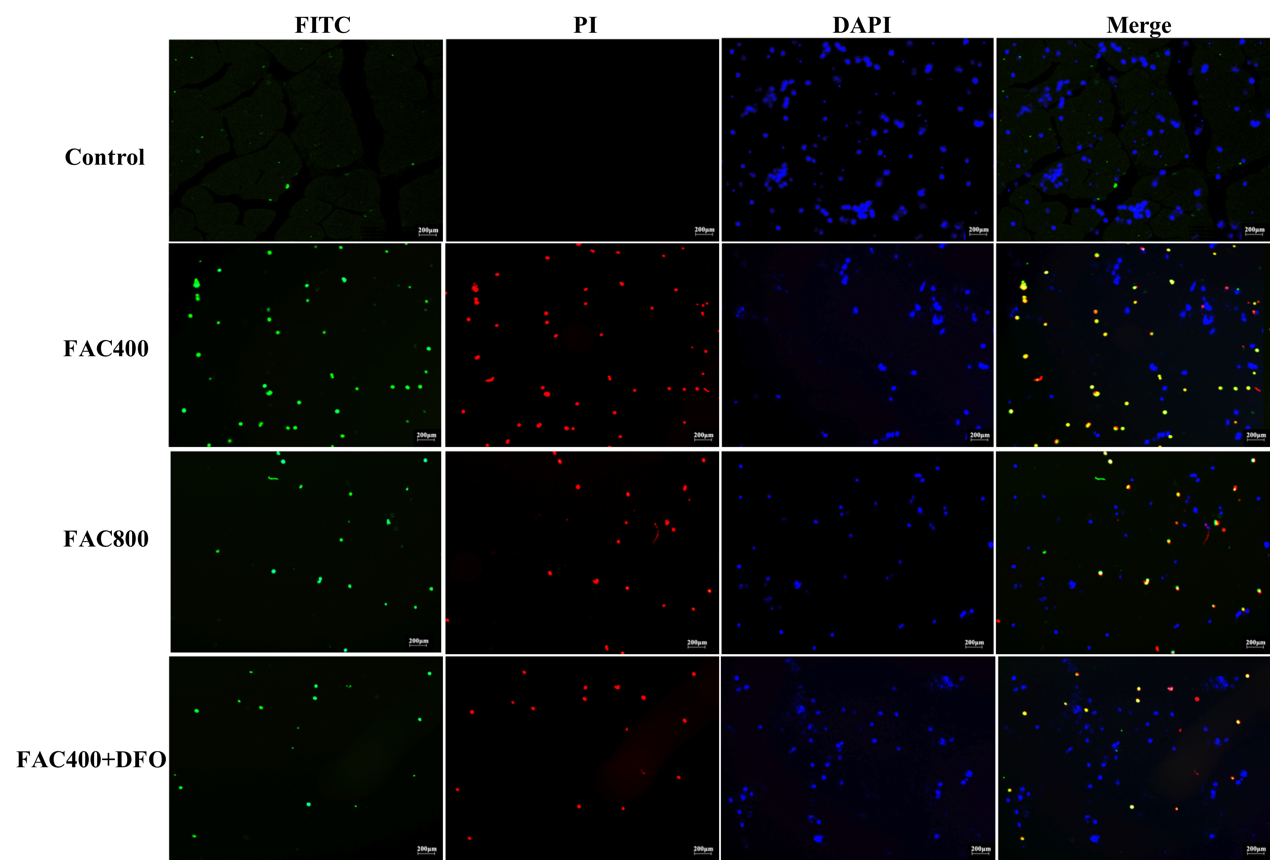


***S Fig3*** *Annexin V-FITC/PI kit was used to assay cell apoptosis rate; Each test was repeated three times and averaged，The difference between each group was compared with Bonferroni method and/or Dunnett’s T3 method. The test level is α=0.05;*P<0.05; ** P<0.01; ** P<0.001*

**Supplementary Figure 4**


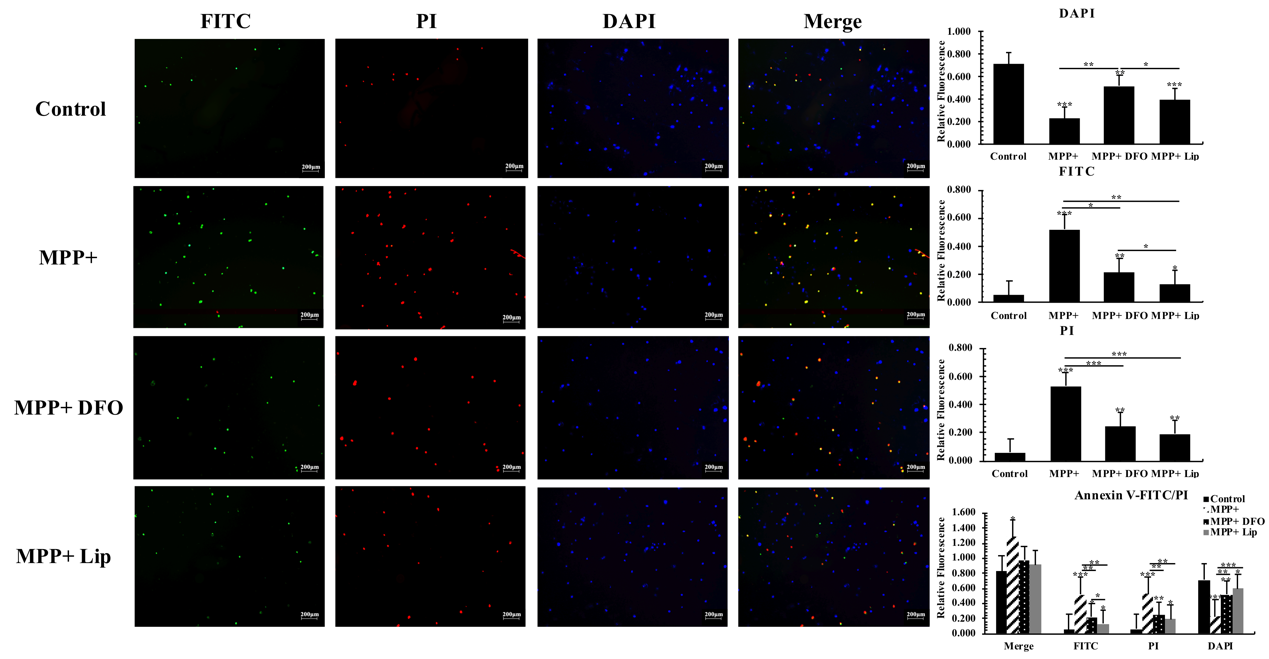


***S Fig.4*** *Annexin V-FITC/PI kit was used to assay cell apoptosis rate; Each test was repeated three times and averaged，The difference between each group was compared with Bonferroni method and/or Dunnett’s T3 method. The test level is α=0.05;*P<0.05; ** P<0.01; ** P<0.001*

***NGF differentiated PC12 Cells***

After treatment with NGF (50 ng/ml), PC12 cells developed obvious synapses (on 2^nd^ day), and the volume of cell bodies increased and changed from circular/elliptical to polygonal/diamond shape. As the duration of stimulation was prolonged, the number and length of cell synapses increased. After 6~8 days, the cell morphology resembled that of neurons (Supplementary **Figure 1A**). In addition, we used a western blot to analyze the expression levels of dopaminergic neuron-specific marker tyrosine hydroxylase (TH). Briefly, compared to day 0, an increase in TH expression was observed on the second day of differentiation (P<0.05). On day 4 and 6, TH expression levels were significantly increased (day0 *vs* day4, *P*=0.016; day0 *vs* day6, *P*=0.001; Supplementary **Figure 1B**).
